# Malignant brain neoplasms incidence and mortality trends in Croatia from 2001 to 2014

**DOI:** 10.3325/cmj.2019.60.33

**Published:** 2019-02

**Authors:** Goran Mrak, Valerija Korent, Ana Mišir Krpan, Andrija Bitunjac, Martina Štenger, Anton Kordić, Hrvoje Barić, Mario Šekerija

**Affiliations:** 1 Department of Neurosurgery, Clinical Hospital Center Zagreb, University of Zagreb School of Medicine Zagreb, Croatia; 2 Department of General Internal Medicine, Gastroenterology, Internistic Oncology, and Hematology, County Hospital Čakovec, Čakovec, Croatia; 3 Department of Oncology, Clinical Hospital Center Zagreb, University of Zagreb School of Medicine, Zagreb, Croatia; 4 Department of Neurosurgery, Division of Surgery, General Hospital Dr. Josip Benčević, Slavonski Brod, Croatia; 5 Department of Pediatric Surgery, Children`s Hospital Zagreb, Zagreb, Croatia; 6 Croatian National Cancer Registry, Division for Epidemiology and Prevention of Noncommunicable Chronic Diseases, Croatian Institute of Public Health, Zagreb, Croatia; 7 Department of Medical Statistics, Epidemiology, and Medical Informatics, Andrija Štampar School of Public Health, University of Zagreb School of Medicine, Zagreb, Croatia

## Abstract

**Aim:**

To analyze the sex-specific incidence and mortality trends of brain malignancies in Croatia from 2001 to 2014.

**Methods:**

Incidence and mortality rates per 100 000 population were calculated using data obtained from the Croatian National Cancer Registry and the Croatian Bureau of Statistics. Rates were age-standardized to the European Standard Population, and trends were assessed using joinpoint regression.

**Results:**

In the observed period there were 6634 new brain malignancy cases (52% men) and 5379 deaths due to this diagnosis (52% men). Age-standardized incidence rates ranged from 9.2-11.5 per 100 000 in men and from 7-8.8 per 100 000 in women. Mortality rates ranged from 7.5-8.7 per 100 000 in men and from 5-6.5 in women. Incidence trends in men, mortality in men, and mortality in women were not statistically significant, while a significant trend was observed in incidence in women (annual percent change -1.5; 95% confidence interval -2.3 to -0.6). No joinpoints were observed in any of the joinpoint analyses by sex for incidence and mortality. Age-specific incidence and mortality rates in both sexes indicate a trend shift toward older age. The proportion of morphologically verified cases ranged from 40.2%-62.4% in men and from 38.6%-56.3% in women; the proportion of death-certificate-only cases ranged from 3.3%-9.4% in men and from 3.3%-17.5% in women.

**Conclusion:**

Incidence and mortality of brain malignancies in Croatia are among the highest in Europe, while reporting on brain malignancies is still poor. There is a need for improved care of patients with brain malignancies and detailed and accurate data reporting.

Although brain malignancies account for approximately 1.8% of all malignancies worldwide (five-year prevalence), some of the histological types are among the most aggressive malignancies (1). Overall, 20% of adults diagnosed with a primary brain malignancy between 2000 and 2007 in Europe survived 5 years after diagnosis (2). Despite increase in one-year relative survival rates in the same period and population, survival remains poor (2). Between 1993 and 2007, the incidence of brain malignancies in most countries worldwide was stable (3).

Brain malignancies are classified according to the WHO Classification of the Tumors of the Central Nervous System (CNS), which was updated in 2016 and includes over 120 entities (4). The incidence and survival for brain malignancies vary significantly by age and histological type (2). Epidemiologic data on brain malignancies are mostly collected from population-based cancer registries, as is the case with Croatia, where brain malignancies in 2014 accounted for 2.2% of all new cancer cases (5). However, imperfections in data collection and inconsistent usage of diagnostic criteria hamper the comparison of epidemiological data across different countries or regions (6).

According to the recent estimates (data for both sexes combined) of the European Cancer Information System for 2018, Croatia ranks fourth in incidence and second in mortality of malignant brain and CNS tumors (7). Thus far, Croatian epidemiologic data trends on brain malignancies have been reported in a publication from 2017, which reported incidence data from 1993-2007 as a part of an analysis of global patterns and incidence (3) and in a series of articles on childhood, adolescents, and young adults’ brain and CNS malignancies as a part of the cooperation of the South-Eastern Europe cancer registries (8-12). Croatian data on brain malignancy incidence have also been included in Volumes IX, X, and XI of the Cancer Incidence in Five Continents publications (13-15). Other epidemiologic data on brain malignancies in Croatia are published in the yearly bulletins of the Croatian National Cancer Registry (5). However, long-term trends in incidence and mortality of brain malignancies in Croatia have not been reported. Therefore, the aim of this study was to provide an overview of these indices from 2001 to 2014.

## METHODS

The incidence and mortality data were obtained from the Croatian National Cancer Registry (CNCR). The CNCR is a population-based cancer registry that collects data on new cases of cancer in the Croatian population. Its data sources include mandatory reports by the primary and secondary health care providers, pathology reports, and death certificates from the Croatian Bureau of Statistics. Brain malignancies were defined according to the ICD, 10th revision, code C71. To calculate incidence and mortality rates per 100 000 population, we used population estimates for years 2001-2014 from the Croatian Bureau of Statistics. The European Standard Population from 1976 was used as a reference when calculating age-standardized rates (ASR) (16).

To describe incidence and mortality time trends of brain malignancies in Croatia, we carried out a joinpoint regression analysis of age-standardized rates using Joinpoint Regression Program, Version 4.6.0.0, from April 2018; Statistical Methodology and Applications Branch, Surveillance Research Program, National Cancer Institute. This approach determines age-standardized cancer incidence and mortality rates along with respective standard errors and fits the simplest model possible, starting with the model with zero joinpoints (17). Using Monte Carlo Permutation method, this approach tests whether adding additional joinpoints (up to a previously defined maximum) is significant. For the period between two joinpoints (or the beginning and the end of series), a log-linear model with annual percent change (APC) is calculated as a trend measure, with its corresponding confidence intervals (CI). The analysis included logarithmic transformation of the rates, standard error for heteroscedasticity, maximum number of two joinpoints, a minimum of two years between two joinpoints, and a minimum of two years between a joinpoint and the beginning or end of the series. A permutation test was used with a significance level set at *P* < 0.05. All tests were two-sided.

## RESULTS

**Figure 1:**
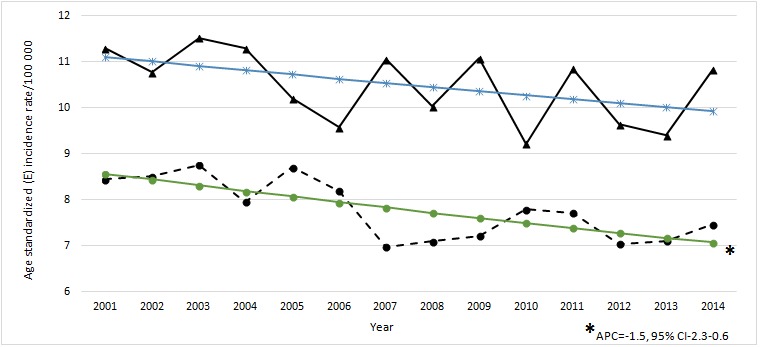
Age-standardized (**E**) incidence rates of brain malignancies for 2001-2014 period, Croatia, by sex. Triangle – men; circle – women; blue line – men modeled; green line – women modeled. E – the European Standard Population.

**Figure 2:**
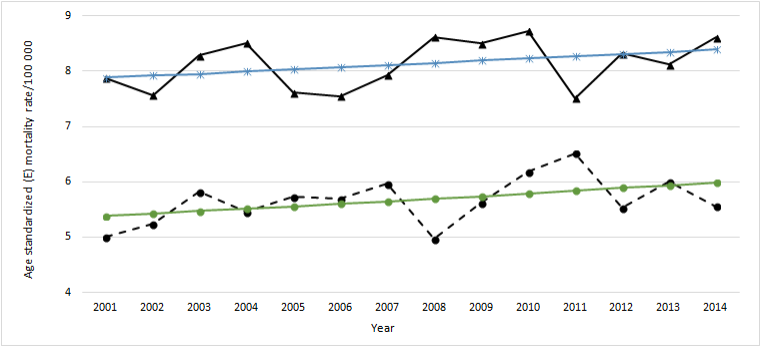
Age-standardized (**E**) mortality rates of brain malignancies for 2001-2014 period, Croatia, by sex. Triangle – men; circle – women; blue line – men modeled; green line – women modeled. E – the European Standard Population.

From 2001 to 2014 there were 6634 reported cases of brain malignancies in Croatia, 52% of which were in men. There were 5379 deaths due to brain malignancies, 52% of which were in men. The number of annually newly diagnosed cases ranged from 439 in 2013 to 506 in 2011. Age-standardized incidence rates ranged from 9.2 to 11.5 per 100 000 in men and from 7 to 8.8 per 100 000 in women; the mortality rates ranged from 7.5 to 8.7 per 100 000 in men and from 5 to 6.5 in women (Table 1). Incidence trends in men (APC -0.9; 95% CI -1.8 to 0.1), mortality in men (APC 0.5; 95% CI -0.3 to 1.3), and mortality in women (APC 0.8; 95% CI -0.2 to 1.9) were not significant, while a significant trend was observed in incidence in women (APC -1.5; 95% CI -2.3 to -0.6) (Figures 1 and 2). No joinpoints were observed in any of the sex-specific regression analyses for incidence and mortality.

**Table 1:** Incidence and mortality from brain malignancies in Croatia, 2001-2014

	Men				Women			
Year	incidence		mortality		incidence		mortality	
	number	ASR* (E)	number	ASR (E)	number	ASR (E)	number	ASR (E)
**2001**	249	11.29	179	7.87	223	8.44	147	5.01
**2002**	240	10.77	176	7.57	233	8.51	162	5.25
**2003**	268	11.52	198	8.28	237	8.75	176	5.83
**2004**	262	11.29	206	8.51	224	7.95	172	5.46
**2005**	237	10.20	182	7.61	251	8.70	181	5.73
**2006**	230	9.57	189	7.55	230	8.19	179	5.70
**2007**	259	11.06	195	7.93	196	6.97	181	5.97
**2008**	243	10.03	212	8.62	207	7.09	165	4.97
**2009**	267	11.08	210	8.50	228	7.22	188	5.63
**2010**	219	9.22	222	8.72	246	7.79	209	6.19
**2011**	262	10.85	192	7.51	244	7.72	225	6.52
**2012**	234	9.65	216	8.32	214	7.04	196	5.54
**2013**	226	9.40	203	8.12	213	7.11	194	6.01
**2014**	267	10.84	222	8.61	225	7.46	202	5.56
**2001-2014**	3463	10.53	2802	8.16	3171	7.82	2577	5.70

*ASR – age-standardized rate; E – the European Standard Population.

Regarding specific entities within this group of diseases, the rates of cases with unknown histologic tumor type ranged from 41% in 2010 to 59% in 2001. Given the imprecise and informative nature of these data, incidence and mortality rates were not calculated by individual pathologic entities.

**Figure 3:**
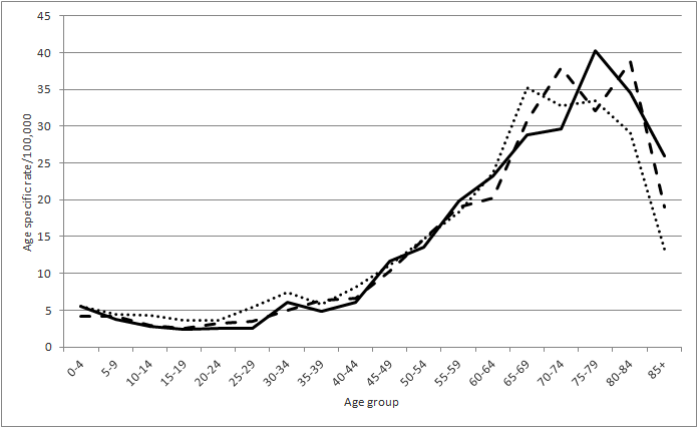
Age-specific incidence rate of brain malignancies in men for different time periods. Dotted line – 2001-2005; dashed line – 2006-2010; full line – 2011-2014.

**Figure 4:**
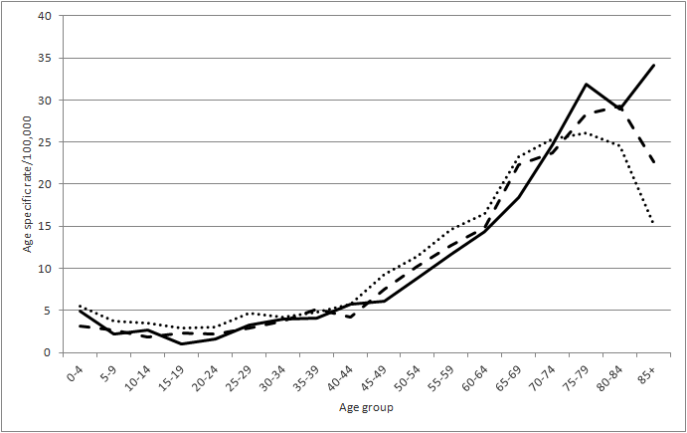
Age-specific mortality rate of brain malignancies in men for different time periods. Dotted line – 2001-2005; dashed line – 2006-2010; full line – 2011-2014.

**Figure 5:**
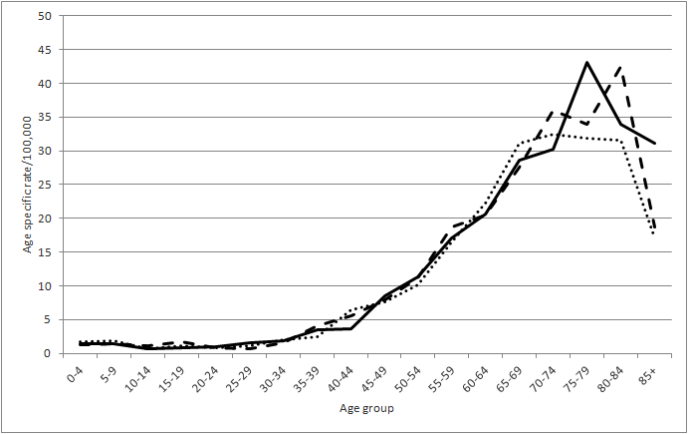
Age-specific incidence rate of brain malignancies in women for different time periods. Dotted line – 2001-2005; dashed line – 2006-2010; full line – 2011-2014.

**Figure 6:**
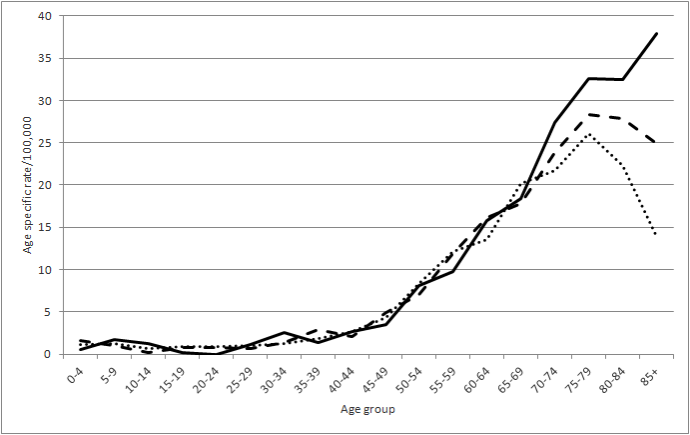
Age-specific mortality rate of brain malignancies in women for different time periods. Dotted line – 2001-2005; dashed line – 2006-2010; full line – 2011-2014.

Age-specific incidence rates of brain malignancies in men, calculated for three separate periods (2001-2005; 2006-2010; 2011-2014), showed a shift in distribution toward old age in the period between 2001 and 2014 (Figure 3). Age-specific mortality rates for men in the same period showed a shift toward younger age, with peak rates in the age group 75-79 (Figure 4). Age-specific incidence and mortality rates in women also showed a shift toward older age, which is particularly evident in mortality rate increase in the age group 85 and older (Figures 5 and 6).

The proportion of morphologically verified cases ranged from 40.2 to 62.4% in men and from 38.6 to 56.3% in women; the proportion of death-certificate-only cases ranged from 3.3 to 9.4% in men and from 3.3 to 17.5% in women (Table 2).

**Table 2:** Registry data quality indicators

Year	Men			Women		
	morphologically verified (%)	death-certificate-only (%)	mortality-to-incidence ratio	morphologically verified (%)	death-certificate-only (%)	mortality-to-incidence ratio
2001	40.2	3.3	0.72	42.6	6.7	0.66
2002	46.6	3.0	0.73	47.0	6.4	0.70
2003	43.8	6.6	0.74	42.7	3.3	0.74
2004	53.1	7.3	0.79	46.0	9.4	0.77
2005	46.3	3.7	0.77	38.6	4.3	0.72
2006	51.3	5.2	0.82	50.6	6.8	0.78
2007	62.4	4.3	0.75	55.7	6.7	0.92
2008	48.8	8.6	0.87	44.5	10.4	0.80
2009	61.0	7.9	0.79	56.3	11.6	0.82
2010	58.5	9.4	1.01	50.6	11.0	0.85
2011	51.5	6.3	0.73	39.4	12.0	0.92
2012	44.9	7.3	0.92	45.1	13.6	0.92
2013	47.5	7.2	0.90	40.6	12.8	0.91
2014	55.1	6.7	0.83	52.8	17.5	0.90

## DISCUSSION

Incidence data obtained in our study ranged from 9.2 to 11.5 per 100 000 in men and from 7 to 8.8 per 100 000 in women, which is in line with the estimates of the incidence of brain and nervous system malignancies from 2018 (1,7) and 2017 (3). In the latter report, age-standardized incidence rate in Croatian men was 12.0 per 100 000, second only to Brazil (13.3), and the incidence in women (9.4) was highest among the included countries (3). This report obtained the incidence data from the national registry, the same source used in this study; however, they included ICD-10 diagnoses C70-C72, while we included only C71 diagnoses. Also, they analyzed the data in 15+ age groups, using truncated incidence rate standardized to the World Standard Population, so the rates are not entirely comparable although they are indicative of the differences between the studied populations. Our study showed a significant decrease in incidence in women, while the report from 2017 found stagnant incidence trends between 1998 and 2007 in the majority of countries (including Croatia, both men and women), apart from decreasing rates in Japanese men (3). There were also countries with a significant increasing incidence trend in men (Thailand, Latvia, Slovenia, Russian Federation, Lithuania) and women (Poland, Latvia, Thailand, Lithuania) (3).

Our study found a slight male predominance in the incidence of brain malignancies (52%). This is in line with the newest global incidence estimates from GLOBOCAN 2018, which reported a higher overall incidence in men (55% vs 45%) (1). We also observed a significant incidence trend in women. GLOBOCAN 2018 report showed that mortality rates in Croatian women were among the highest five and in men among the highest thirteen in the world (18). Armenia had the highest standardized mortality rate for women (5.0 per 100 000) and North Macedonia for men (9.4 per 100 000). Given these facts, the mortality rates in both Croatian women and men remain among the highest globally, and the absence of a decreasing mortality trend alongside the decreasing incidence trend, ie, increased mortality-to-incidence ratio (M/I), raises the question of data validity. This finding is important given that the M/I ratio is sometimes used as a proxy of efficacy of cancer control programs (19) in the absence of reliable population-based survival data and with the good quality of incidence and mortality data. The increased M/I is an alarming finding and warrants research into its causes and caution in interpretation of the results of this study.

For instance, in the absence of a confirmed histological diagnosis, the reliability of primary brain malignancy diagnosis is low, and some of these lesions might not have been primary brain malignancies but some other lesions. The reliability and different standards in collecting data on brain malignancies are a well-known problem in cancer registries worldwide (7).

Our data also have to be cautiously interpreted as the results of the largest study on cancer survival in the world, CONCORD-3, showed that 5-year net survival for patients diagnosed with primary brain malignancies in Croatia in 2010-2014 period was one of the highest in Europe (42%) (20). Furthermore, data from the EUROCARE-5 study for Croatian patients diagnosed in 2000-2007 were excluded from the analysis (along those for Denmark, Finland, Sweden, Northern Ireland, Malta and some regional registries from Italy, Spain and Poland) due to poor data availability and reliability (2). Five-year survival from childhood CNS tumors in Croatia, analyzed separately within the EUROCARE-5 study, was almost 70%, with some indicators of overestimation (21). Very high M/I ratio and a very good survival are in obvious discrepancy, which is why further high-resolution studies are needed to elucidate these findings. They could be partly explained by the quality of mortality statistics, ie, by erroneous coding of malignancies metastatic to the brain as primary brain malignancy (22). This is very relevant since, among European countries, Croatia has one of the highest incidences of other malignancies that are prone to metastasize to the brain (23,24). In any case, urgent efforts are needed by both health professionals and policy makers to improve the quality of care and reporting on brain malignancies, and to conduct further research on the strengths and caveats of the existing data.

The age distribution of brain malignancies and deaths is in accordance with the literature data (1,15). The findings of an age shift toward older age (85+) in incidence and mortality could indicate changes in diagnostic procedures and treatment, but without a more specific insight into trends by histological subgroup this remains a speculation.

A significant decreasing incidence trend in women could also be interpreted as an improvement in the coding of primary cancer site in the cancer registry, however, since no similar changes were observed in men and the mortality trends did not decrease, this explanation seems less plausible. A logical next step in evaluating the cancer control regarding brain malignancies in Croatia would be a retrospective study evaluating the medical histories of patients with brain malignancies using both hospital sources and cancer registry data. Also, all stakeholders should collaborate more closely to improve the collection of morphological diagnoses for brain malignancies. This could facilitate studying of brain cancer trends by histological type, which is currently hampered by the low quality of data indicators in the Croatian National Cancer Registry.

The research in epidemiology of brain malignancies is relatively scarce, and this is the first study to simultaneously quantify the incidence and mortality trends of brain malignancies in Croatia. Our analysis, however, revealed significant gaps in the quality of the existing data. To improve the quality of trend estimates of brain malignancies in Croatia, all medical professionals included in the mandatory collection of data, both for the cancer registry and official mortality statistics, should deliver complete, timely, and reliable documents. Further research, using linkage between existing data sources, could guide future interventions with more precision.
